# Safety, pharmacokinetics, and pharmacodynamics of 4-hour intravenous infusion of eritoran tetrasodium in healthy Japanese and Caucasian males

**DOI:** 10.1186/cc9685

**Published:** 2011-03-11

**Authors:** Y Okubo, N Aikawa, M Lynn, DP Rossignol, YN Wong, E Schuck, Y Kitahara, T Nakano, O Sivak, KM Wasan, C Nagy

**Affiliations:** 1 Eisai, Inc., Woodcliff Lake, NJ, USA; 2 Emergency and Critical Care Medicine, School of Medicine, Keio University, Tokyo, Japan; 3 Eisai, Inc., Andover, MA, USA; 4 Eisai Co., Ltd, Tokyo, Japan; 5 The University of British Columbia, Vancouver, Canada

## Introduction

Activation of TLR4 signaling by endotoxin is believed to be a primary mediator of sepsis and septic shock, via excessive production of cytokines and proinflammatory mediators [1]. Eritoran tetrasodium (hereafter eritoran), a synthetic analog of the endotoxin constituent lipid A, binds to the TLR4/MD-2 complex and thereby blocks the interaction of endotoxin with TLR4 [2]. Eritoran is being investigated for the treatment of severe sepsis [3]. We report results of a study conducted to assess the single-dose safety and tolerability, as well as pharmacokinetics and pharmacodynamics, of eritoran infusion in Japanese and Caucasian healthy adult males.

## Methods

This was a double-blind, randomized, single-center, placebo-controlled, ascending single-dose, sequential-group study. Sixty-four subjects (aged 20 to 45 years; BMI 18 to 30 kg/m^2^) were randomized to four groups: 4 mg total dose (*n *= 12); 12 mg total dose (*n *= 24); 28 mg total dose (*n *= 12); placebo (*n *= 16). Adverse events were recorded by the investigator. Laboratory assessments included standard hematology and clinical chemistry, lipid analysis, and urinalysis.

## Results

There were no serious adverse events. Eritoran in single doses up to 28 mg over 4 hours was well tolerated, with no apparent ethnic differences noted. Plasma concentrations were slightly higher, while clearance and volume of distribution were lower, in Japanese versus Caucasian subjects; these differences were not significant after adjustment for differences in body weight. The *ex vivo *endotoxin inhibitory activity of eritoran was similar in Japanese and Caucasian subjects. Eritoran was distributed mainly to the HDL fraction in both Japanese and Caucasian subjects.

## Conclusions

Eritoran was safe and well tolerated in healthy Japanese and Caucasian subjects. The data do not indicate any need for clinical dose adjustment for possible ethnic-based differences in drug distribution or metabolism.
